# Measurement and Analysis of Interconnects’ Resonance and Signal/Power Integrity Degradation in Glass Packages

**DOI:** 10.3390/mi16010112

**Published:** 2025-01-20

**Authors:** Youngwoo Kim

**Affiliations:** Department of Semiconductor System Engineering, Sejong University, Seoul 05006, Republic of Korea; youngwoo@sejong.ac.kr; Tel.: +82-2-3506-3408

**Keywords:** glass packages, interconnects, measurement, resonance, signal/power integrity

## Abstract

In this article, resonance phenomena of high-speed interconnects and power delivery networks in glass packages are measured and analyzed. The resonances are generated in the interconnection by the physical dimension, cancelation of reactance components, and modes. When the resonances are generated in the operation frequency band, the signal/power integrity of the interconnect can be affected. As such, resonances generated in high-speed interconnects increase insertion loss, which degrades signal integrity. Also, resonances of the power delivery network (PDN) associated with boundary conditions increase PDN impedance, which degrades power integrity by generating power/ground noise and return current discontinuity of through vias. Recently, glass packaging has been gaining more attention due to its advantages associated with low substrate loss and large dimensions compared to silicon wafers. However, the low loss of the substrate and process variation may affect the resonance properties of interconnects. The resonance impacts on signal/power integrity must be analyzed, and mitigation plans should be proposed to maximize the advantages of the glass packaging technology. To analyze the resonance impacts on signal/power integrity, various glass package test vehicles are designed and fabricated. The fabricated test vehicles include transmission lines, PDNs, and patterns to measure an interaction between the through via and PDN. First, transmission line patterns that have 50-ohm characteristic impedance are measured. Due to the process variations, quarter-wave resonances are monitored, and at those frequencies, a sharp increase in insertion loss is observed, which deteriorates the signal integrity of the interconnect. Various PDN patterns are measured in the frequency domain, and regardless of the PDN shape, PDN impedance peaks are observed at the mode resonance frequencies. Due to a low-loss characteristic of the glass substrate, sharp PDN impedance peaks are generated at these frequencies. Also, at these frequencies, both signal and power integrity degradations are measured and analyzed. To fully benefit from the advantages of glass packaging technology, a thorough electrical performance analysis should be conducted to avoid resonances in the target frequency range.

## 1. Introduction

The semiconductor industry is entering the post-Moore’s law era represented by system scaling enabled by advanced packaging technologies. Especially, chiplet and advanced interconnection technologies are driving such trends, enabling customizable 2.5-dimensional systems [1,2,3,4]. A chiplet is a compact, modular integrated circuit (IC) designed to perform a specific function, which can be combined with other chiplets, ICs, and even high-bandwidth memory (HBM) [5,6] on a package to realize highly customizable and high-performance semiconductor systems. The new standard of chiplet, universal chiplet interconnect express (UCIe) [7], mandates advanced package design rules and strict signal/power integrity requirements to ensure extremely high signal bandwidth of the system. As such, if advanced packages are adopted, the package should be carefully designed to satisfy 1317 GB/s/mm signal bandwidth.

In the market, silicon interposers are widely adopted as an advanced package to integrate HBMs and graphic processing units (GPUs) to form a high-performance 2.5-D graphics card. The silicon interposer is capable of increasing the integration density and the number of high-speed channels since it enables fine pitch metallization [8]. Because of these advantages, 2.5-D integration based on silicon interposer technology increases the system bandwidth significantly [9]. Even though silicon interposers are widely adopted, there are critical issues associated with limited wafer dimensions (12 inches) and additional manufacturing processes mandated to isolate the conductors from the silicon substrate with finite conductivity. Recently, due to artificial intelligence (AI) applications and data center servers, more HBMs, chiplets, and system on chips (SoCs) are expected to be integrated on advanced packages [10]. The current silicon interposer, which has a limit to increase the dimensions, is expected to meet the recent integration trends and suffer from cost issues associated with fabrication yield related to the wafer dimension. Also, the conductivity of the silicon substrate can cause significant signal integrity (SI) issues [11] above 16 GHz, which is the Nyquist frequency of the chiplet [7].

Glass packaging technologies are proposed as a superior alternative to silicon interposers. The glass substrate and polymers for build-up layers have several advantages: excellent dimensional stability, a closely matched coefficient of thermal expansion (CTE) to various ICs with silicon substrates to be assembled, availability of glass substrates in large and thin panel sizes compared to that of silicon wafers, and excellent electrical resistivity of the glass substrate that allows for low signal loss up to the GHz range [12,13]. The electrical resistivity of the glass substrate ensures low insertion loss, which is an important criterion for maintaining the signal integrity of high-speed channels. The glass packaging is gaining interest once again, recently driven by Intel for more powerful computing performance [14]. Recent trends driven by artificial intelligence applications, data center servers, and big data require large computing power and system bandwidth. As a result, more HBMs, SoCs, and chiplets must be integrated on advanced packages for a small form factor, and the signal/power integrity of interconnects should be maintained to ensure the performance. Therefore, 2.5-D customizable integration based on large-scale glass packages is a potential means of achieving high-bandwidth systems with large integration density.

**Figure 1:**
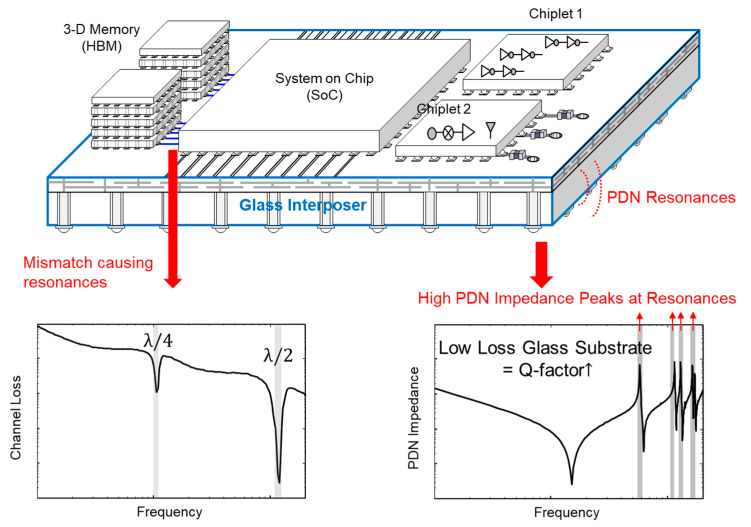
Impacts of resonances on signal and power integrity of interconnections in the glass package are conceptually depicted. When resonances are generated, signal loss (channel loss) increases and signal integrity is degraded. Also, resonances generate PDN impedance peaks affecting power/ground noise and return current of vias.

Maintaining the signal/power integrity is key to realizing a high signal bandwidth mandated by the standard [7]. The low-loss glass substrate is suitable for high-speed signaling, but resonances of the high-speed channel and power delivery network (PDN) should be carefully analyzed. The resonances are generated in the interconnection by the physical dimension, cancelation of reactance components, and modes. When the resonances are generated in the operation frequency band, the signal/power integrity of the interconnect can be affected [15,16]. The low loss of the substrate and process variation may affect the resonance properties of interconnects in glass packages. Figure 1 depicts resonance impacts that affect signal/power integrity. As shown in Figure 1, signal loss of the channel can increase when the resonance is generated in the high-speed channel, which degrades signal integrity. Usually, PDNs have larger physical dimensions than high-speed interconnects, and mode resonances can be generated within the PDNs due to current and voltage boundary conditions. At mode resonance frequencies, a dramatic increase in the PDN impedance profile is observed, as depicted in Figure 1. The high PDN impedance deteriorates the signal/power integrity since it generates large power/ground noise, return current discontinuity of the through via, return current loading to the PDN, and electromagnetic interference (EMI) radiation. Therefore, resonances affecting signal/power integrity of high-speed channels and PDNs in the glass package must be analyzed, and mitigation plans must be discussed.

In this article, resonance phenomena of high-speed interconnects and power delivery networks in glass packages are measured and analyzed. It is important to verify and report the resonances degrading signal/power integrity of the interconnects in glass packages. To analyze the resonance impacts on signal/power integrity, various glass package test vehicles are designed and fabricated. The fabricated test vehicles include transmission lines, power delivery networks (PDNs), and patterns to measure an interaction between the through glass via (TGV) and PDN. First, transmission line patterns that have 50-ohm characteristic impedance are measured. Due to the process variations, quarter-wave resonances are monitored, and at those frequencies, a sharp increase in insertion loss is observed, which deteriorates the signal integrity of the interconnect. The impact of quarter-wave resonance has been continuously reported in PCB designs, but it is measured and reported for the first time in this article for the glass package channel.

To verify and report resonances of the glass package PDNs degrading both signal and power integrity, various PDN patterns are designed, fabricated, and characterized. The test vehicles include power/ground planes, a meshed structure to satisfy the metal density rules in advanced packages, a PDN with an embedded decoupling capacitor, and a PDN-TGV interaction pattern. Regardless of the PDN shapes, mode resonances are measured above the GHz range. At these frequencies, PDN impedance peaks are measured, and these impedance peaks are different from other advanced packages [17]. Due to a low-loss characteristic of the glass substrate, sharp PDN impedance peaks are generated at these frequencies. Also, at these frequencies, both signal and power integrity degradations are measured and analyzed. PDN impedance peaks can be suppressed by the on-chip PDN capacitance or embedded capacitors within the glass package PDN, but they are effective for partially suppressing the PDN impedance or shifting the resonance peaks. To fully benefit from the advantages of glass packaging technology, a thorough electrical performance analysis should be conducted to minimize or to avoid resonance impacts on signal/power integrity of interconnects.

## 2. Designed and Fabricated Test Vehicles to Measure the Resonance Impacts on Signal/Power Integrity

This section explains the fabrication processes of the glass package test vehicles. Depending on applications, glass packages may have different numbers of layers, single- or double-sided designs, and even the existence of through glass vias (TGVs). However, for advanced packages, TGVs and microvias must be adopted for higher system integration density. Also, the double-sided lamination on both sides of the glass substrate provides dimensional stability and, at the same time, provides better electrical design flexibility. Therefore, double-sided glass packages are designed and fabricated, including the glass substrate and two metal build-up layers on top and bottom of the glass substrate. To verify and report resonances affecting the signal integrity in high-speed interconnects of the glass package, microstrip lines and coplanar waveguide (CPW) lines are designed, fabricated, and measured. However, these test vehicles do not require TGVs, so the top side of the glass substrate is used to realize the test vehicles. To analyze resonances affecting both the signal and power integrity in glass packages, PDN patterns and TGV channels penetrating PDNs are designed, fabricated, and characterized. In these test vehicles, TGVs are required to interconnect measurement pads and PDNs. Both PDNs and TGV channels are characterized to analyze PDN-TGV interactions affecting both the signal and power integrity.

Most of the fabricated test vehicles have a total of four metal layers, except for the microstrip line pattern, which only has two metal layers at the top side of the glass substrate. The glass substrate may vary between 50 and 300 μm in thickness depending on its application, but the 100 μm thick glass substrate (EN-A1) is adopted for this study to design and fabricate the test vehicles. Through vias are drilled and polymer layers (organic dry films: ZS-100) are applied. The build-up dielectric has superior properties such as low dielectric loss similar to that of the glass substrate, a smooth surface, and ease of panel processing. The average roughness of the build-up layers after the desmear process is controlled to enable fine-line patterning and to reduce the signal loss above the GHz range due to skin effects. The low CTE, similar to the silicon and high modulus, prevents warpage and enables substrate flatness after each fabrication process. Freshly drawn glass substrate itself is regarded as a very rigid material. However, the through glass via (TGV) formation and dicing processes induce defects in the glass, which affect the strength of the glass substrate. The low-loss polymer layers laminated on both sides of the glass substrate are crucial for forming metal layers that make the package functional, and at the same time, they prevent the substrate from cracking [18,19].

**Figure 2:**
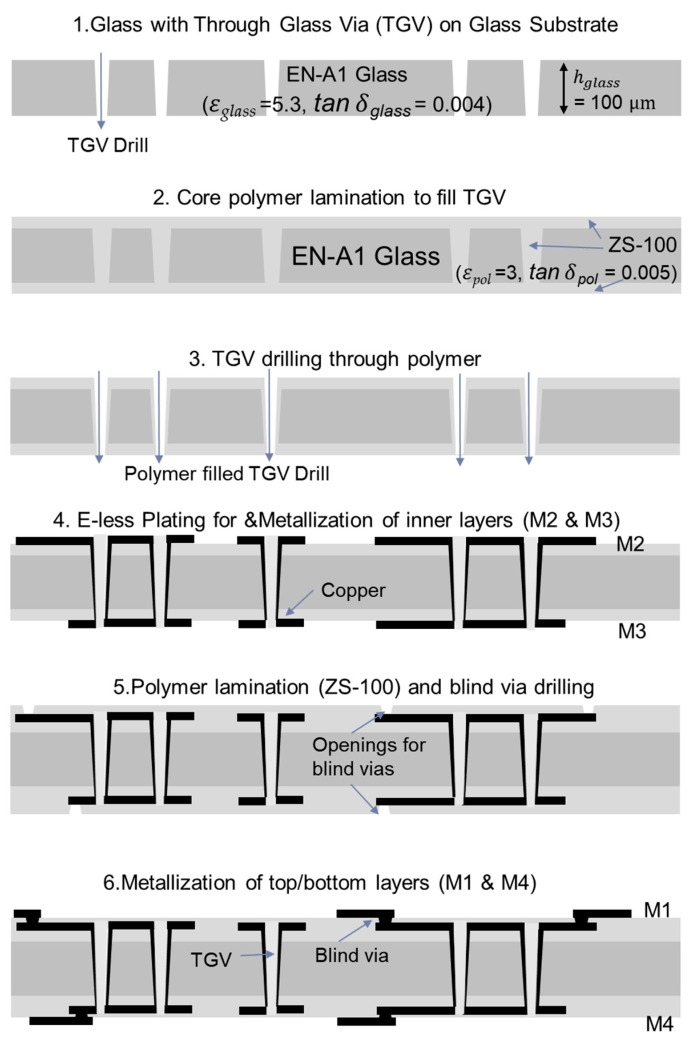
Fabrication processes of the glass package are depicted by showing laminates and cross-sections.

Figure 2 shows the cross-section of the glass package test vehicles and briefly explains the fabrication process steps. The glass substrates are processed at a 6 inch × 6-inch (15.2 mm × 15.2 mm) panel size. The fabrication processes can be scaled to 500 mm × 500 mm to accommodate more chiplets and HBMs. Such scalability has a potential for increasing the fabrication yield and high-volume manufacturing. The glass package fabrication begins with the through vias’ formulation. After cleaning the glass substrate, silane coupling agents are applied to coat both sides of the glass substrate for polymer layers’ lamination. The dry-filming process is conducted simultaneously for both sides by applying a vacuum hot-press at 100 °C for 60–90 s followed by curing at 130 °C for 60 min. The polymers (ZS-100 laminated on top/bottom of the glass substrate) attain low viscosity during the curing process that allows them to flow and completely fill the through vias. Laser ablation is applied to the vias filled with polymer to reopen the vias at a slightly smaller diameter to accommodate the alignment tolerance of the laser-drilling system. Recently, direct metallization on the glass substrate is being tried without polymer layers, but these layers actually provide several benefits. As such, enhanced handling of the large-scale glass substrates, stress buffering between metal patterns and the glass substrate for improved reliability, moisture absorption protection, and maintaining the signal loss.

The coated polymer layers aid in high-throughput electro-less (E-less) copper seed layer deposition onto the substrates, which is crucial for the metallization process [20]. Ultraviolet (UV) lithography and semi-additive processing (SAP) are adopted to metalize the inner circuitry layers M2 and M3 with precision. The same lamination and SAP processes are repeated to form the outer metal layers (M1 and M4). In between the inner and outer metal layers, microvias are formed.

**Figure 3:**
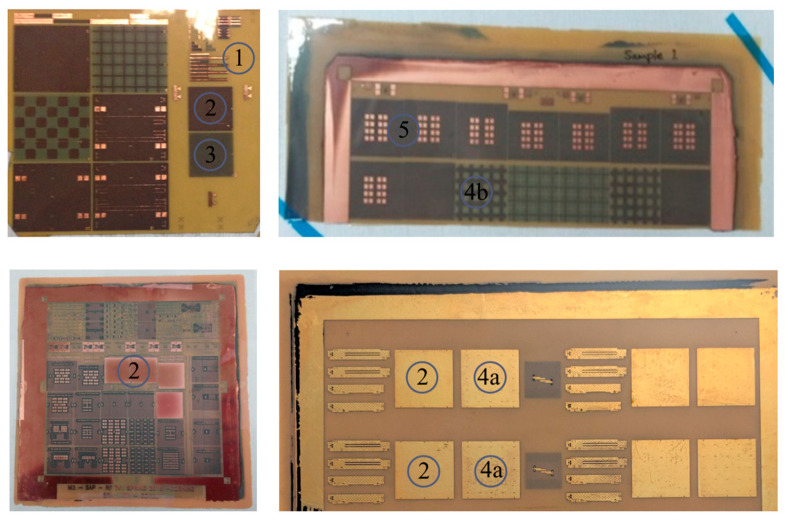
Fabricated glass package coupons are shown. Each coupon includes various test vehicles to be measured and analyzed.

Figure 3 shows fabricated glass package coupons including various test vehicles to be measured and analyzed. Table 1 summarizes test vehicles included within coupons. Each coupon has slightly different design parameters and dimensions, but similar test vehicles are inserted to verify the design in various ways, such as electromagnetic (EM) simulation and process variation comparison. Detailed explanations, such as top-view and cross-sectional view of each test vehicle, will be given in Section 3 and Section 4 of this article.

## 3. Experimental Validation of Process Variations Generating Resonances of the High-Speed Channel in Glass Packages

**Figure 4:**
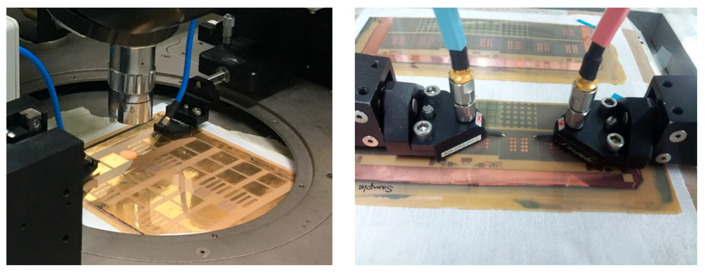
Test vehicles are measured on two different probe stations depending on coupon sizes with microprobes, coaxial cables, a vector network analyzer, and an oscilloscope.

In this section, microstrip lines and CPW lines in test vehicle 1 are measured to analyze the interconnects’ resonance impacts on signal and power integrity. As can be seen in Figure 4, various test vehicles are measured on two different types of probe stations depending on the dimensions of the coupons. Microprobes (Picoprobe GSG type with 250 μm pitch, GGB industries Inc., Naples, FL, USA), calibration kit (#CS-9, GGB industries Inc.), and coaxial cables (W.L. Gore & Associates, Inc., Newark, DE, USA) for both time and frequency domain measurements. The frequency domain measurements are conducted using the vector network analyzer (VNA) up to 20 GHz (N5230A from Agilent Technologies, Santa Clara, CA, USA). In the frequency domain, insertion loss of the channel is measured. The definition of insertion loss and measurement methods are summarized in [21,22]. To measure in the time domain, a pulse-pattern generator (PPG) model MP-*1763C* from Anritsu (Atsugi, Japan) and a digital sampling oscilloscope model TDS800B from Tektronix (Beaverton, OR, USA) are used. In the time domain, reflected voltage waveforms are measured and analyzed to determine the characteristic impedance of interconnect.

**Figure 5:**
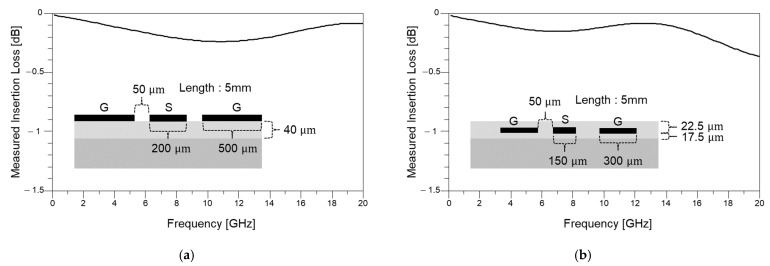
Measured insertion losses of coplanar waveguide (CPW) lines designed to have 50-ohm characteristic impedance. (**a**) CPW realized in M1 layer and (**b**) CPW realized in M2 layer are measured. For both cases, resonances are not observed in the measured frequency range.

Fifty-ohm-matched transmission lines are designed, fabricated, and measured. First, coplanar waveguide (CPW) lines are designed on the M1 and M2 layers. Since both lines have different effective permittivity characteristics, the dimensions to realize 50-ohm matching are different. Three-dimensional EM simulations are conducted to decide the dimensions of the CPW lines. Figure 5 shows measured insertion losses of both cases. The CPW line realized in M1 can be directly measured using the GSG-type microprobe. Additional microvias and pads should be designed and fabricated on M1 to measure the CPW line realized in M2. Figure 5a shows the measured insertion loss of the CPW line in M1, and Figure 5b shows the measured insertion loss of the CPW line in M2. In both cases, it has been evaluated that the interconnects are well fabricated with controlled process variations resulting in maintained 50-ohm characteristic impedance (variation within 10%, minimum of 47-ohm time domain reflectometer (TDR) impedance measured). Since the characteristic impedances are controlled for both cases, resonances are not monitored in the measured results. Since the case shown in Figure 5b has inevitable impedance mismatches at pads and microvias, more loss and fluctuation in the measured insertion loss are observed.

**Figure 6:**
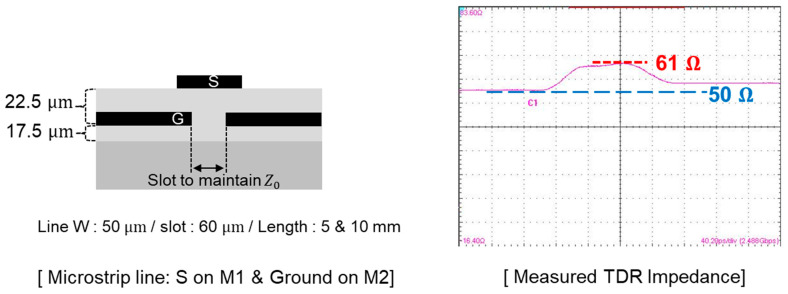
The designed microstrip line on the M1/M2 layer is fabricated. To maintain 50-ohm characteristic impedance considering process/design rules, a slot must be realized in M2. Due to over-etching during the fabrication process, measured TDR impedance is higher than the tolerance range.

Next, the microstrip line is designed, fabricated, and measured. Figure 6 shows the cross-section of the designed microstrip line and measured TDR impedance. The signal line is designed in the M1 layer, and it has a width of 50 μm. For such a case, it is impossible to realize the 50-ohm characteristic line by applying the simple equation $Z_{0}=\sqrt{\frac{L}{C}}$. The capacitance of the microstrip line had to be decreased to satisfy the 50-ohm characteristic impedance. As a result, the slot in the ground plane in the M2 layer is designed to reduce the electric field formed in between the signal line and ground plane. Using the 3-D EM simulator, the slot width is determined to be 60 μm. However, additional design steps may increase the possibility of process variations. The TDR impedance of the fabricated microstrip lines is measured, and the result is shown in Figure 6. The goal is to maintain the characteristic impedance of the microstrip line 50 ± 5 ohm, but over-etching has occurred during the M2 layer fabrication process. As a result, the capacitance between the signal line and ground plane has been reduced more than expected, resulting in a higher characteristic impedance of the interconnect.

**Figure 7:**
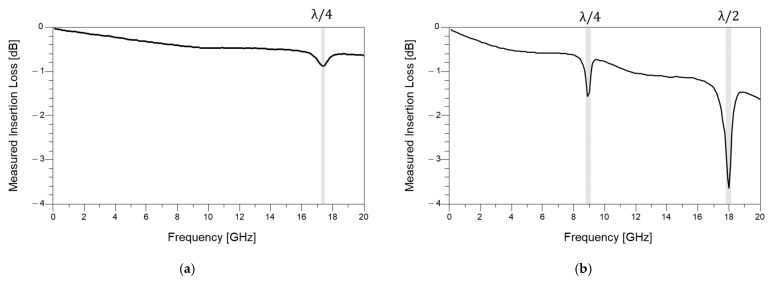
Measured insertion losses of microstrip lines. (**a**) Length of 5 mm and (**b**) Length of 10 mm lines are measured, respectively. Quarter-wave resonance impacts on insertion loss profiles are observed. At these frequencies, insertion loss increases dramatically.

In Figure 7, measured insertion losses of the fabricated microstrip lines are plotted. For both cases shown in Figure 7a,b, insertion loss peaks are generated at certain frequencies. These frequencies are related to the quarter-wave resonance [23]. Quarter-wave resonances are thoroughly analyzed in the RF/PCB design field, but their impact on glass packages has not been discussed. When impedance mismatch or discontinuity occurs, insertion loss increases dramatically at the quarter-wave resonance frequency and its harmonics. In the case of analogue or RF applications, these frequencies must be carefully estimated and avoided near the operation frequency. If the operation frequency and insertion loss peaks coincide, signal communication or operation of the system can be severely degraded. In the case of digital signaling, eye diagrams are used to judge the signal integrity. The eye diagram is determined by the broadband response of the signal, especially harmonics of the frequency related to the signal’s data rate. If such peaks exist within the insertion loss profile of the interconnect, signal quality can be severely degraded.

**Figure 8:**
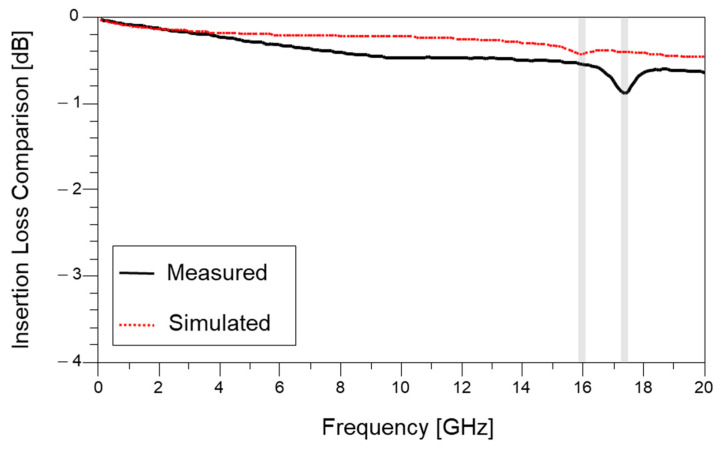
Comparison between measured and simulated insertion losses. Simulation is capable of estimating the existence of quarter-wave resonance frequencies within the frequency range, but accurate estimation is limited.

It is important to estimate the existence of quarter-wave resonances within the frequency range of interest. Therefore, 3-D EM simulations must be conducted, but most of the 3-D EM simulations are conducted assuming the designed parameter itself. When designing the channel of advanced packages, process variations or at least design parameter case studies must be conducted to confirm the existence of the resonance. In Figure 8, a comparison between measured and simulated insertion losses is shown. Three-dimensional EM simulation is capable of estimating the existence of quarter-wave resonance frequencies within the frequency range, but accurate estimation is limited since it is difficult to estimate the range of process variations and the occurrence location within the interconnect. If resonance impacts are expected, channel dimensions must be carefully redesigned to avoid the resonance within the target frequency range to minimize signal integrity degradation.

## 4. Glass Package PDN Impedance Peaks Measurement and SI/PI Degradation

In this section, fabricated glass package PDNs are measured and analyzed. Test vehicles 2, 3, 4a, and 4b are measured. In the PDNs, modes associated with boundary conditions (current flow at edges is zero) dominate the PDN impedance profile. PDN impedances at the mode resonance frequencies are measured and analyzed.

### 4.1. Measured PDN Impedance Characteristics

**Figure 9:**
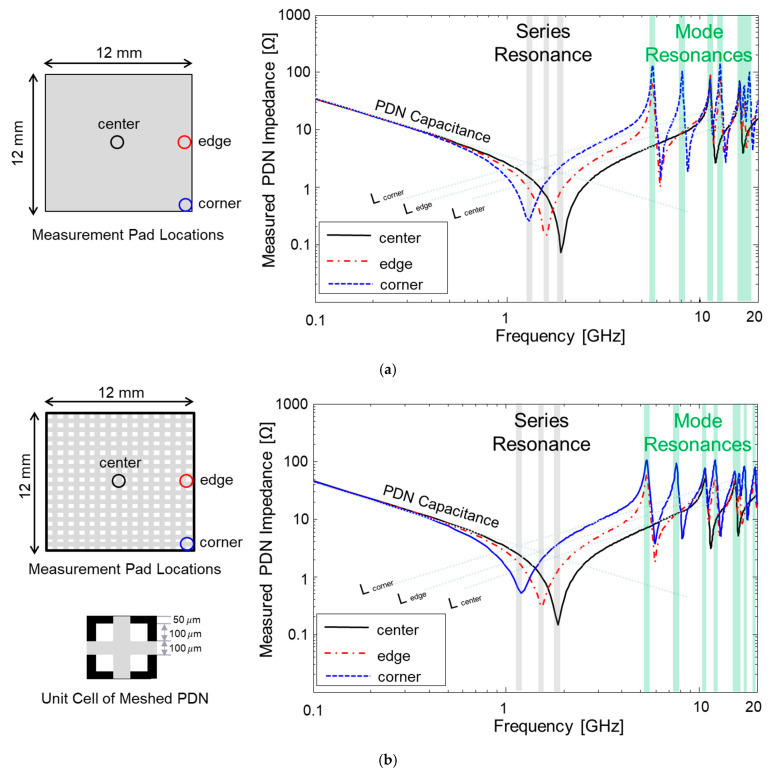
Measured PDN impedances are plotted: (**a**) PDN impedances of plane-type PDN; (**b**) PDN impedances of meshed-type PDN.

In this section, fabricated glass package PDNs are measured and analyzed. PDN impedances of plane-type and meshed-type PDNs are plotted in Figure 9.

Plane-type PDNs (power plane on M1 and ground plane on M3) are measured at the center, edge, and corner sides. Using the VNA, the reflected waves are measured and formulated to Z-parameters to plot the PDN impedances in the frequency domain. The results are plotted in Figure 9a. Regardless of measurement locations, the PDN capacitance, which dominates the low-frequency range, remains the same. However, the PDN inductances are affected by the current loop size. As a result, the corner side of the PDN experiences the largest inductance. As a result, the series resonance frequency is lowest for the case measured at the corner. Above series resonance frequencies, mode resonances generate PDN impedance peaks. Mode resonance frequencies for rectangular PDNs can be estimated based on Equation (1):

(1) $$f_{mn}=\frac{c}{2\sqrt{\varepsilon_{PDN}}}\sqrt{{\left(\frac{m}{l_{x-PDN}}\right)}^{2}+{\left(\frac{m}{l_{y-PDN}}\right)}^{2}}$$

In Equation (1), *m* and *n* represent mode numbers, *c* is the speed of light, $\varepsilon_{PDN}$ is the effective permittivity of the PDN (glass substrate and polymer layers), and $l_{x-PDN}/l_{y-PDN}$ are physical dimensions of the PDN in both the *x* and *y* axes. Mode resonances will be formed at the lower frequency range if the PDNs with larger dimensions are designed in the glass package. For arbitrary or irregular shared PDNs, 3-D EM simulation is required to accurately estimate mode resonance frequencies of the PDN. In the PDNs, modes associated with boundary conditions (current flow at edges is zero) dominate the PDN impedance profile above the series resonance frequency. In Figure 9b, measured PDN impedances of meshed-type PDNs are plotted, and it shows a similar PDN impedance profile compared to results shown in Figure 9a. Regardless of the PDN shape, mode resonances affect the PDN impedance profile. High PDN impedance peaks cause power/ground noise ($∆V_{power/ground\;noise}=I_{circuit}\times Z_{PDN}$), which degrades the power integrity of the glass package [24].

**Figure 10:**
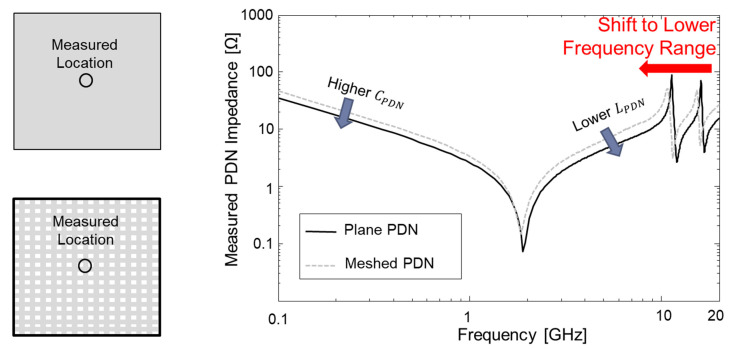
Comparison between measured PDN impedances of plane PDN and meshed PDN (both measured at the center of the PDN).

In Figure 10, a comparison between measured PDN impedances of the plane-type PDN and meshed-type PDN is shown. It is apparent that the meshed structure has smaller PDN capacitance compared to that of the plane structure. As a result, the PDN impedance of the plane-type PDN shows a slightly lower impedance profile than that of the meshed-type PDN. In the case of the meshed PDN, the current loop becomes larger than that of the plane PDN. As a result, the plane PDN shows a lower impedance profile above the series resonance frequency. Mode resonance frequencies shift to a lower frequency range for the meshed-type PDN.

**Figure 11:**
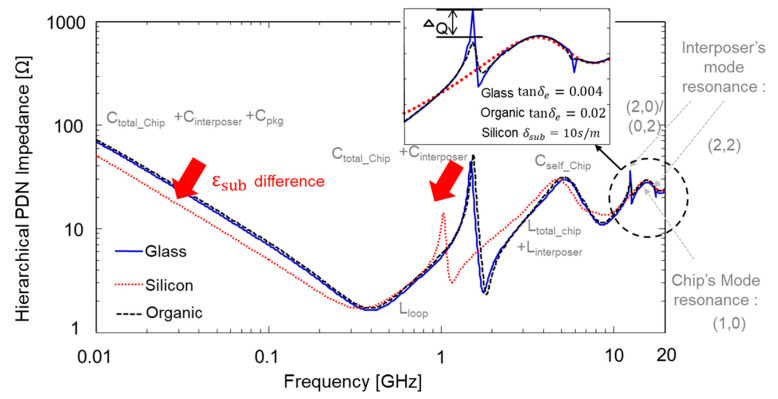
Comparison of hierarchical PDN impedances assuming the same condition except the substrate material of the advanced package (interposer) [16].

It is important to suppress the PDN impedance peaks associated with mode resonances since high PDN impedance profiles generate large power/ground noise [24] and cause return current discontinuity of through via channels penetrating the PDN [25]. PDN impedance peaks associated with mode resonances are generated at relatively high frequency ranges. To suppress such impedance peaks, adding relatively smaller capacitance compared to the board-level decoupling capacitors is useful. On-chip PDN capacitance or embedded package substrate (EPS) capacitors can be a powerful solution. In Figure 11, hierarchical PDN impedance profiles are compared with different interposer (advanced package) PDN materials assuming the same board and on-chip PDNs [16]. As can be seen in Figure 11, on-chip capacitance significantly suppressed the impedance peaks associated with the mode resonances, but they cannot be completely suppressed. Also, the glass substrate, which has the lowest substrate loss, shows the largest PDN impedance peaks.

**Figure 12:**
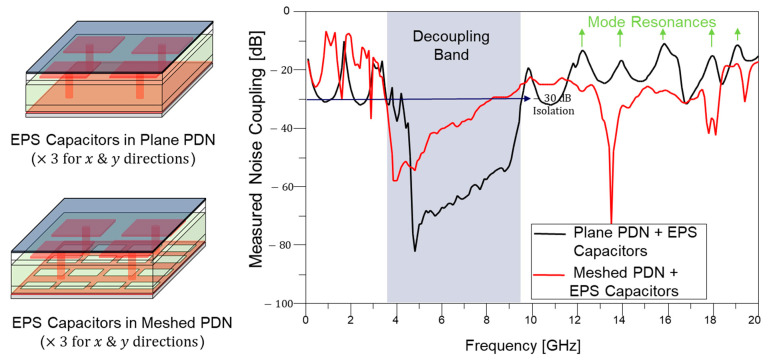
Measured noise couplings with embedded package substrate (EPS) capacitors in the plane PDN and meshed PDN are compared. Still, impacts of mode resonances increasing the noise coupling are observed.

In Figure 12, the measured noise couplings with embedded package substrate (EPS) capacitors in the plane PDN and meshed PDN are compared. A total of 16 embedded decoupling capacitor arrays are inserted in both the plane PDN and meshed PDN. As a result, a noise decoupling band is created, and it is marked in Figure 12 as well. The EPS capacitor array reduces both PDN self-impedance and transfer impedance ($Z_{21}$ or $S_{21}$ in the PDN). However, as can be seen from Figure 12, impacts of mode resonance still exist, which increase the noise coupling in the PDN. These impacts are even maximized when the through via penetrates the PDN, which will be discussed in the following subsection. It is extremely difficult to fully suppress mode resonances in the PDN. Therefore, the existence of PDN impedance peaks associated with mode resonances must be estimated, analyzed, and proper solutions should be adopted. As such, adding decoupling capacitors (embedded capacitors in package substrates or on-chip decoupling capacitors) with smaller capacitance compared to decoupling capacitors in the PCB shifts mode resonance frequencies outside the target frequency range determined by circuits’ data rates.

### 4.2. PDN Modes Affecting the Signal Integrity and Mitigation Method

In this subsection, both signal and power integrity degradation associated with mode resonances in glass packages are measured, analyzed, and reported. PDN impedance peaks associated with mode resonances become a source of power/ground noise. The mechanism of the interaction is shown in this subsection. The electrical design of glass packages mandates SI/PI co-analysis to avoid various resonances for both high-speed interconnects and PDNs.

**Figure 13:**
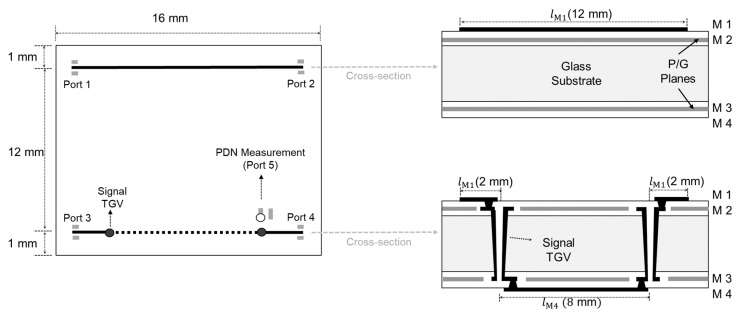
Explanation of a test pattern measuring PDN and signal interactions. TGV channel is penetrating the PDN.

The designed and fabricated test vehicle to measure the interaction between signal TGVs and PDNs is described in Figure 13 (test vehicle 5 in Table 1). It shows the top and cross-sectional view of the test vehicle. Measurements are conducted in three steps, and port numbers are shown in Figure 13:

Step 1: Measure insertion losses ($S_{21}$ and $S_{43}$) in the frequency domain;

Step 2: Measure $S_{55}$ and convert to $Z_{55}$, compare with $S_{43}$;

Step 3: Measure eye diagrams between ports 1–2 and ports 3–4.

**Figure 14:**
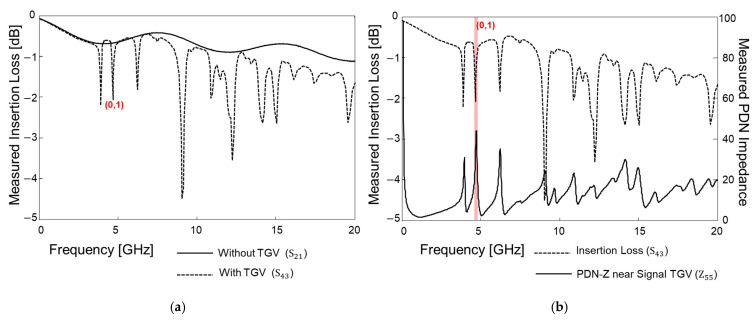
(**a**) Insertion losses are measured and compared without and with TGVs penetrating the PDN. (**b**) Insertion loss of the channel with TGV transitions is compared with the PDN impedance measured near the signal TGV. The (0,1) mode resonance frequency is analyzed in the time domain as well.

In Figure 14, measurement results are plotted and compared. The insertion losses of the interconnect without and with TGV transitions are compared in Figure 14a. The insertion losses are measured up to 20 GHz, and the insertion loss profiles are similar for both cases except for some frequencies for $S_{43}$. These insertion loss peaks are not associated with quarter-wave resonances; they are related to the PDN impedance peaks associated with mode resonances of the PDN. The PDN impedance ($Z_{55}$) near the signal TGV has a low impedance profile up to 20 GHz, but high-impedance peaks are generated at the mode resonance frequencies. At these frequencies the return current of the signal TGV is severely affected and discontinued by the mode resonances rather than other PDN factors. At the mode resonance, the return current of the channel with TGVs is affected directly since the high-impedance PDN loads more power than the receiver. The low loss of the glass substrate generates sharp PDN impedance peaks affecting the return current of the TGV channel. Because of this reason, the return current is discontinued, and the insertion loss increases significantly. At these frequencies, signal integrity can be severely deteriorated.

**Figure 15:**
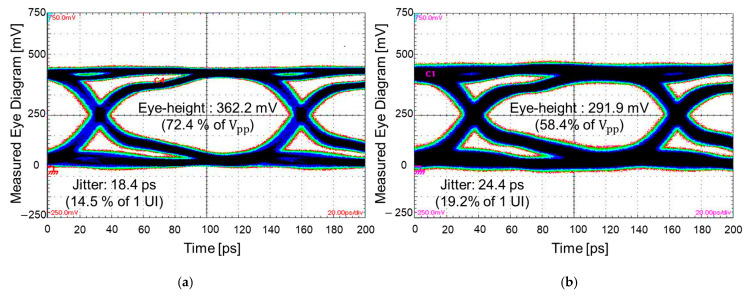
Eye diagrams are measured and compared. The data rate corresponds to the frequency of the (0,1) mode shown in Figure 14b: (**a**) eye diagram without TGV; (**b**) eye diagram with TGV transitions.

Measured eye diagrams are plotted and compared in Figure 15. A pseudo-random binary bit sequence (PRBS) of $2^{8}-1$, with a rise/fall time of 30 ps and an input data rate that corresponds to the (0,1) mode, is injected into the channel without and with TGVs penetrating the PDN. To generate such input waveforms, the PPG is used at the transmitting sides (ports 1 and 3), and an oscilloscope is used at the receiver side (ports 2 and 4) to measure and plot the eye diagram. Eye diagrams are obtained by superimposing received digital signals and are often used to judge the signal integrity of the high-speed interconnect. At this frequency (corresponding to mode (0,1) of the PDN), as can be seen in Figure 14a, insertion loss increased dramatically for the channel with TGVs. As a result, the eye diagram shown in Figure 15b is worse than the eye diagram shown in Figure 15a. At the PDN, more resonance frequencies and signal integrity degradation are measured and observed in both the frequency and the time domain. To mitigate such impacts associated with PDN mode resonances, the impedance peaks must be suppressed, or the TGV channel should be properly shielded with decoupling capacitors or ground TGVs.

It has been experimentally validated that there are various types of resonance that exist in the advanced packages. The resonance is affected by various factors such as the dimension of interconnects, material properties, process variations, and signal/power interactions. Each mechanism should be understood to suppress the resonance impacts on the signal/power integrity of interconnects to fully benefit from the advantages of the glass packaging technology. Especially, low loss of the glass substrate ensures signal integrity associated with maintained insertion loss up to the GHz range. As a result, the glass packaging technology is gaining interest once again as a superior substitute to silicon interposers, showing various limits associated with the loss of silicon substrate and the wafer dimension affecting the yield. However, as reported in this article, glass packages also suffer from signal integrity degradation due to resonances. Also, low loss of the substrate can deteriorate both signal and power integrity in some cases. Therefore, thorough analysis of various resonances is mandatory when designing the glass package to maximize the advantages and potential.

## 5. Conclusions

In this article, resonance phenomena of high-speed interconnects and power delivery networks in glass packages are measured and analyzed. The resonances are generated in the interconnection by the physical dimension, cancelation of reactance components, and modes. Each resonance has different impacts on the signal/power integrity of interconnects. To understand the mechanism and impacts of the resonance in the glass package, various test vehicles are fabricated and measured. The fabricated test vehicles include transmission lines, power delivery networks (PDNs), and patterns to measure an interaction between through vias and PDNs. First, transmission line patterns that have 50-ohm characteristic impedance are measured, such as CPW lines and microstrip lines. Due to the process variations, quarter-wave resonances are monitored, and at those frequencies, a sharp increase in insertion loss is observed, which deteriorates the signal integrity of the interconnect. Various PDN patterns are measured in the frequency domain, and regardless of the PDN shape, PDN impedance peaks are observed at the mode resonance frequencies. Due to a low-loss characteristic of the glass substrate, sharp PDN impedance peaks are generated at these frequencies. Also, at these frequencies, both signal and power integrity degradations are measured and analyzed. To fully benefit from the advantages of glass packaging technology, a thorough electrical performance analysis should be developed to avoid resonance and mitigate techniques.

## Figures and Tables

**Table 1:** Test vehicles and numbering marked in Figure 3 with material properties.

	Symbol	Purposes	Description
Test vehicles and purposes	1	SI degradation measurement	Transmission line patterns (Microstrip lines and CPW lines)
	2	PDN-Z measurement	Plane-type PDN (12 mm × 12 mm)
	3	PDN-Z measurement	Meshed PDN (12 mm × 12 mm)
	4a	PDN-Z measurement	PDN with embedded decoupling capacitor
	4b	PDN-Z measurement and PI characterization	PDN with embedded decoupling capacitor and mesh/defects
	5	SI/PI degradation	PDN-TGV interaction validation pattern
Material properties	ε *_glass_*		5.3 at 2.4 GHz
	ε *_pol_*		3 at 10 GHz
	tan δ *_glass_*		0.004 at 2.4 GHz
	tan δ *_pol_*		0.005 at 10 GHz
	Copper conductivity (metallization)		$5.8\;\times \;10^{7}\;\sigma /m$

## Data Availability

The original contributions presented in this study are included in the article. Further inquiries can be directed to the author.
